# A Dictionary of Treatment, or Therapeutic Index

**Published:** 1892-06

**Authors:** 


A Dictionary of Treatment, or Therapeutic Index, including Medical and Surgical Therapeutics. By William Whitla, M. D., Professor of Materia Medica and Therapeutics in Queen's College, Belfast. Revised and adapted to the Pharmacopeia of the United States. In one octavo volume of 917 pages. Cloth, $4.00. Philadelphia: Lea Brothers & Co. 1892.

Of late, many books have been issued that are specially adapted to ready reference by the busy physician, some of which have been works of value, while others are only of indifferent worth. This book of Whitla belongs to the better class, and will be found one of the most satisfactory of its kind. It is not a work that can be given an analysis in these pages, but we commend it to the profession as one of the foremost reference books that have lately been put forth.

